# Online Appointment Scheduling for a Nuclear Medicine Department in a Chinese Hospital

**DOI:** 10.1155/2018/5148215

**Published:** 2018-04-11

**Authors:** Qian Xiao, Li Luo, Shu-zhen Zhao, Xiao-bin Ran, Ya-bing Feng

**Affiliations:** ^1^Institute of Hospital Management, West China Hospital, Sichuan University, Chengdu 610041, China; ^2^Service Management Institute, Business School of Sichuan University, Chengdu 610064, China; ^3^Outpatient Department, West China Hospital, Sichuan University, Chengdu 610041, China

## Abstract

Nuclear medicine, a subspecialty of radiology, plays an important role in proper diagnosis and timely treatment. Multiple resources, especially short-lived radiopharmaceuticals involved in the process of nuclear medical examination, constitute a unique problem in appointment scheduling. Aiming at achieving scientific and reasonable appointment scheduling in the West China Hospital (WCH), a typical class A tertiary hospital in China, we developed an online appointment scheduling algorithm based on an offline nonlinear integer programming model which considers multiresources allocation, the time window constraints imposed by short-lived radiopharmaceuticals, and the stochastic nature of the patient requests when scheduling patients. A series of experiments are conducted to show the effectiveness of the proposed strategy based on data provided by the WCH. The results show that the examination amount increases by 29.76% compared with the current one with a significant increase in the resource utilization and timely rate. Besides, it also has a high stability for stochastic factors and bears the advantage of convenient and economic operation.

## 1. Introduction

Because of the persistent combination of rapid demand and slow supply, “difficulty and high cost to access medical service” has become one of the hot social issues in China, which draws extensive attention in the study of medical services. Nuclear medicine, a subspecialty of radiology, playing an important role in the proper diagnosis and timely treatment of diseases, has its unique problem in patient appointment scheduling. In the process of nuclear medical examination, short-lived radiopharmaceuticals (drugs that give off radiation) produced by special generators are administered to take high-quality images within the body with the help of ionizing radiation, and improper scheduling probably leads to its unavailability. Therefore, hospital managers are under a great pressure to manage such special resource efficiently and effectively.

This problem is very critical in large tertiary hospitals which are heavily overloaded. For example, the demand for nuclear medicine in the West China Hospital (WCH) which is one of the largest single-site hospitals in the world, with 4,300 inpatient beds, and the major referral center for complex health problems for Southwestern China, grows rapidly at a rate of nearly 20% a year, intensifying the conflict between supply and demand of medical resources. One solution is to increase resource supply, such as purchasing more equipment. However, due to their high fixed cost and operation cost, expensive equipment has been strictly controlled by the authorities to control healthcare costs. Furthermore, according to our investigation, the current scheduling strategy based on experience does not make full use of resources. For instance, under the condition of current scheduling strategy, the contradiction of supply and demand of two key resources (equipment and medicine) involved in the process of nuclear medical examination is prominent. During a week, the bottleneck of nuclear medical examination shifted from equipment to medicine. From Monday to Tuesday, excessive production of medicine resulting in low medicine utilization (the average utilization rate is only 64.1%) causes an equipment bottleneck. From Wednesday to Friday, however, insufficient medicine production leads to poor efficiency of the resource use (idle equipment and staff) and low patient satisfaction. In this case, medicine becomes another bottleneck. Furthermore, at weekends, the fluctuation of the patient flow makes the resource utilization instable. Therefore, applying scientific methods to achieve appointment scheduling of radiology resources at the Nuclear Medicine Department in the WCH has important applicable significance.

Patient requests in nuclear medicine arrive in an online fashion during the day as the scheduling proceeds, which makes it unique with very limited research reported in the literature. Stochastic planning techniques are an alternative to address this problem. However, unlike common online problems considering only one kind of resource, such as devices working hours or plane capacity, this one is more complex under multiple resource constraints within the time window originating from the short half-life of the radiopharmaceuticals. Adopting a method similar to that of Pérez et al. [1], we solve this problem through two steps: determining the offline optimal value (the offline scheduling) and the reasonable online scheduling strategy (online scheduling). In the first step, based on the known arrival sequence, we develop a nonlinear integer programming model considering the characteristics of the Nuclear Medicine Department, such as multiresources constraints and time window constraint, and apply a multipopulation genetic algorithm (MPGA) for its solving. In the second step, as the overall time sequence is unknown, the optimum solution of the previews step cannot guarantee global optimum. We apply stochastic online algorithms to solve stochastic integer programs.

The contributions of this paper are as follows. First, we develop a nonlinear integer programming model considering the characteristics of the nuclear medicine department, such as multiresources constraints and time window constraint, and get optimal solution by MPGA. Second, we design a stochastic online algorithm considering the demands of various types of patients and arrival unpredictability, which is close to the greatest level of reality by removing many assumptions in previous studies, such as the risk-neutral and predicable assumption about the patient demands.These contributions will help the practice of nuclear medicine by providing increased patient throughput and higher utilization of resources and will improve the methodology of the appointment scheduling in nuclear medicine.

The rest of this paper is organized as follows.  Section 2 reviews related research works on this problem and summarizes the application of online algorithms in the Nuclear Medical Department. The nuclear medicine scheduling problems are described and corresponding solutions are proposed in Section 3.  Section 4 provides a nonlinear integer programming model in offline scheduling and genetic algorithms for its solving. The intractability and sensitivity analysis of this problem are also carried out in this section. An optimal dynamic scheduling strategy is proposed and a series of simulation experiments are conducted in Section 5. The paper ends with some concluding remarks and directions for future research in Section 6.

## 2. Literature Review

As healthcare expenditures and demand have been rising dramatically worldwide, increasing attention from many academicians and practitioners has been paid to the efficiency of health service, such as medical personnel staffing, medical resource allocation, and appointment scheduling. Appointment scheduling is an important determinant of efficiency, timely access to health services, and patient satisfaction [2]. In recent years, medical appointment scheduling has grown comprehensively in the literature, including outpatient scheduling [3–5], surgery scheduling [6–8], and medical examination scheduling [9–11]. Regarding the review papers [2, 12], the appointment scheduling system can be regarded as a queuing system, of which the simplest case is when all scheduled patients arrive punctually in their appointment times and a single doctor serves them with stochastic processing times. Factors like multiple patients, servers and service, presence of unpunctual patients, no-shows, walk-ins, and emergencies make the scheduling problem more complicated. Regarding the limited existing literature on the nuclear medicine scheduling problem, similar research approaches are taken. Green et al. [13] solved the scheduling problem for different types of patients with random arrival in the MRI Department. Taking it as a finite-horizon dynamic schedule problem that allows only one patient to arrive at each slot where a single sever is available and only one patient can be served at a time, they obtain some properties related to the optimal strategy. Based on this study, Patrick et al. [14] set different priorities among patients, modeled the scheduling process as a Markov decision process, and then solved the equivalent linear program through approximate dynamic programming, for the state space is too large for a direct solution. Kolisch and Sickinger [15] worked on a similar problem. Different from former researches, they considered two CT scanners and compared the decision rules under different schedule strategies. They made a qualitative leap in theory for two servers, although the two are taken from an identical machine. Wu et al. [16] designed scheduling rules considering the diversity of examination tasks and the different levels of facilities and the simulation results show that allocating the tasks in this way can fix the service time, balance the medical task among several resources, and improve the utilization rate. Akhavizadegan et al. [17] applied a finite-horizon Markov decision process as dynamic programming to formulate a scheduling problem in a nuclear medical center by considering the patients' choice behavior and different no-show rates for patients. Seeking to reduce the potential impact of delays on radiation therapy cancer patients, Sauré et al. [18] studied scheduling practices at a local hospital. Formulating a discounted infinite-horizon Markov decision process for scheduling cancer treatments in radiation therapy units, using an affine architecture to approximate the value function, and adopting the column generation method to solve the equivalent linear programming model, they obtained an approximate optimal strategy. Liu and Geng [19] considered two different examinations, two urgency levels, patients' no-shows, and physicians' overtime and proposed a discount-cost Markov decision process (MDP) with the objective of maximizing the expected revenue from examining patients and minimizing the overtime penalty. In a word, most published papers regard the nuclear medicine scheduling problem as a common one without considering the use of radioactive isotopes with short half-life, which makes it a unique problem within the time window. Furthermore, multiple resources, such as medicine and equipment, are required in the procedure. However, to our knowledge, few published papers have considered such factor in patient scheduling in this field.

Regarding the literature, there are two common approaches to schedule an appointment, namely, offline scheduling and online scheduling. The offline scheduling approach, static [12] or allocation [17] scheduling approach, makes decisions at the beginning of planning and scheduling and allocates known demand to available resources, whereas the online scheduling approach, dynamic [12] or advanced [17] scheduling approach, revises continuously during the scheduling period based on the current state of the system and assigns resources to the patient in advance of the service date. As the most common appointment scheduling problem in healthcare, the offline problem is the most concentrated on by most of the literature. To the best of our knowledge, there are few researches applying online algorithms in healthcare, especially in nuclear medicine departments, despite having good application and fruitful theory achievement in various areas, especially in manufacturing and services. For example, Karhi and Shabtay [20] studied an online scheduling problem of two job types on a set of multipurpose machines aiming at minimizing the makespan. Ma et al. [21] studied the online scheduling of linear deteriorating jobs on a single machine to minimize the total weighted completion time. Ni and Xu [22] considered the joint ticket pricing and booking problem in the airline industry from the perspective of online algorithms and competitive analysis and proposed policies which can dynamically adjust price and allocate the tickets according to the set of bookings previously offered at any point in time. Referring to the literature, one of the advantages of online scheduling system is that it can be adjusted according to the practical situation and bring about better resource utilization. Ball and Queyranne [23] pointed out that although the static strategy can guarantee an optimal competitive ratio, it is more realistic to adjust the strategy according to the actual demand. Moreover, for patient requests in nuclear medicine arriving in an online fashion during the day as the scheduling proceeds, it is more suitable to apply stochastic planning techniques to address the appointment scheduling problem. Our work is inspired by Pérez et al. [1]. They used the online algorithm for appointment scheduling in the nuclear medicine considering the multiresource constraints and time window characteristics. They divided the operation time in a day into time slots of equal length and assumed that patients' appointment times coincide with the beginning of a time slot and patients show up for their appointment most of the time. Under these conditions, they built an integer programming model which can be directly solved by a linear programming solver. We also considered multiresource and time window constraints and proposed a nonlinear integer programming model which is solved by the multipopulation genetic algorithm (MPGA) in the offline scheduling, but only two key resources, medicine and equipment, are focused on and examination items are divided into four groups accordingly, whereas in Eduardo's study, they analyzed several resources such as technologists, nurses, gamma cameras, and sometimes a treadmill in each procedure performed in nuclear medicine with their current procedural terminology (CPT) codes. Considering real situations in Chinese hospitals, our model may be a good tradeoff between feasibility and scientificity. Furthermore, our scheduling system is easier to be used and understood by a practicer, usually a nurse in the nuclear medicine department, with limited computer operation skills, compared to Pérez et al.'s NMOS (nuclear medicine online scheduling) and NMSOS (nuclear medicine stochastic online scheduling) algorithms that must be implemented in JAVA and ILOG CPLEX.

In conclusion, the characteristics of multiresources constraints and specific time window in nuclear medicine constitute a unique problem with very limited research reported in the literature. Moreover, there is hardly any research in radiology appointment scheduling applying online algorithms considering patient's demand and arrival distribution. For this reason, this paper not only has guiding significance for nuclear medical departments in practice, but also is a supplement to the theory.

## 3. Problem Definition and Notation

### 3.1. Resource Constraint

The practice in the Nuclear Medicine Department of the WCH can be divided into two categories: treatment and examination. The latter includes PET/CT and SPECT/CT. We focus on SPECT/CT for its heavy use. The resource constraints include equipment and medicine. Technetium and iodine are the two major medicines involved in SPECT/CT scans (see Table 1). I131 is not taken as a constraint for its long half-life and easy access, whereas Tc99 imposes constraints on appointment scheduling. It is produced by two molybdenum generators purchased from another place once a week. Due to the decay of molybdenum with a half-life of only 67.2 h, the production of Tc99 decreases exponentially within a week. We take three pieces of equipment available for SPECT/CT scans as identical machines for their tiny efficiency variance and being used for similar examination item.

Examination items in the Nuclear Medicine Department of the WCH are up to 24 types. Among them, whole body bone scan, kidney scan, thyroid scan, salivary glands scan, I131 therapeutic agent scan, bone dynamic scan, assessment of glomerular filtration rate (GFR), and I131 whole body scan account for 95.59%, while the rest account for less than 5%. For this reason, the top eight are studied and then are divided into four groups according to similar resource consumption and injection method (see Table 2).

### 3.2. Problem Description

Compared with other kinds of online scheduling problems, this one has its unique characteristics by considering the multiresources constraints and the time window. A similar scheduling problem has been deeply researched in the field of revenue and production management, though only one resource constraint (e.g., device working time or plane capacity) is considered. In the nuclear medicine department, two key resource constraints are involved, which makes the scheduling more difficult. In addition, this scheduling problem has a time window of 1–3 days. On the one hand, for the timely examination being the premise of timely medical practices and care, the examination date is fixed within 3 days after their arrival day; on the other hand, patients will not be examined on the arrival day due to the particularity of nuclear medicine.

## 4. Offline Scheduling Model

### 4.1. Nonlinear Integer Programming Model

In the offline problem, all patient requests are assumed to be known in advance. Thus, patient scheduling is made by considering all requests for the day. Considering multiresources and time window constraints, we propose a nonlinear integer programming model aiming at maximizing the weekly examination amount while satisfying all constraints. A set of patient requests (*x*_*gpd*_) is used as input to the model. Each patient *p* requests an examination in group *g* on day *d* with binary value (1 for acceptance, 0 for denial).

The notations required are listed in Scheduling Problem Sets and Parameters, and the offline scheduling model is

(1a) max ∑g∈G ∑p∈P ∑d∈Dxgpd,

(1b)  ∑d∈Dxgpd≤1,g∈G,  p∈P;

(1c)  ∑g∈G ∑p∈Ptg∗xgpd≤td,d∈D;

(1d)  ∑g∈G ∑p∈Pkg∗xgpd≤kd,d∈D;

(1e)  wl≤d∗∑d∈Dxgpd−agp≤wug∈G,  p∈P;

(1f)  xgpd  is  the  binary.

The objective function of (1a) maximizes the weekly examination amount. Constraint (1b) enforces examination for each patient to be at most once a week to avoid overlapping. Constraint (1c) limits the working time of equipment for each day. Constraints (1d) enforce the medicine amount for each day. Constraint (1e) is the time window constraints which make sure the examination day is within a certain period after the arrival day.

### 4.2. The Offline Model Solving

Being an NP problem, the 0-1 integer programming model, a typical model extensively used in operations research, is hard to be solved. This paper adopts a more complex nonlinear integer programming model with a large scale. Therefore, the solution for this model is more complicated and differs substantially from the general one. Thus, this problem cannot be solved by common methods used in general integer linear programming models which may be theoretically possible, like branch and bound method or enumeration method. For the same reason, neither can software like Cplex.

Due to the dramatic expanding search space, it is difficult or even impossible to get optimal solution by enumeration method in large-scale problem solving, so researchers turn to a satisfactory one. Genetic algorithm (GA) is one of the best tools for satisfactory solution with advantages like good convergence, low computational complexity, high robustness, and so forth. Practices show that the GA is quite effective in the nondeterministic polynomial complete problem (NPC) solving. For this reason, we apply the genetic algorithm to solve this problem.

#### 4.2.1. Standard Genetic Algorithm

**Figure 1:**
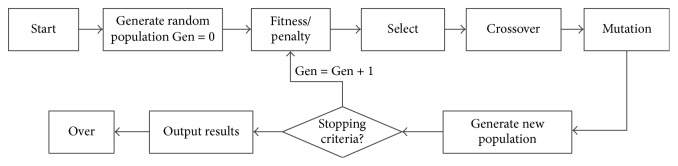
The logic diagram of the algorithm.

*(1) Algorithmic Design and Implementation*. The logic diagram of the algorithm is shown in Figure 1. 

Here, the floating-point coding method is adopted for not producing larger redundant space and being good at satisfying complicated constraints of decision variables in large space searching, compared to the binary encoding method. The string length of chromosomes is set as the weekly demand and is divided into 28 parts, each of which is for one type of patient decided by arrival day and examination item. Each position on the chromosome is for an unprocessed demand and is expressed numerically as 0–7. 0 is for denial and 1–7 is for what day of the week the examination day is. Since the demand is about 600 a week, the solution space will be up to 8^∧^600 (2^∧^1800). Fitness is represented by objective function value, but to better satisfy all the constraints, the evaluation function of individual fitness is adjusted by penalty value to limit functions which do not meet all the restrictions. Roulette selection is used as a selection operator, two-point crossover is adopted as crossover operator, and adaptive mutation and basic bit mutation are introduced as mutation operators.

*(2) Parameter Setting*

*Equipment Constraint*. Based on a real situation, working hours are set to about 10 h a day.

*Medicine Constraint*. Medicine production varies from day to day within a week as in Table 3.

*Time Window Constraint*. The examination day is fixed within 1–3 days after the arrival day.

*Arrival Rate*. Data analysis indicates that most of the data obey Poisson distribution by K-S test while only a few do not but can be fit by uniform distribution, so we take stochastic arrival sequence as an input as in Table 4.

*(3) Results*. The problem is solved by GA program on Matlab with a total running time of 721 s and the optimum of 576. The rest of the other results are shown in Table 5.

#### 4.2.2. Multiple Population Genetic Algorithm

**Figure 2:**
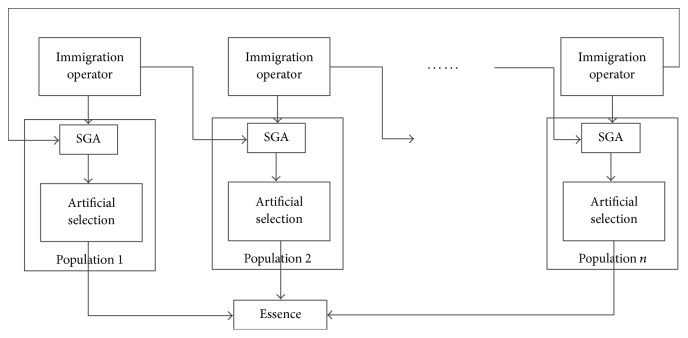
The logic diagrams of multipopulation genetic algorithm.

Aiming at solving the existing problems of standard genetic algorithm (SGA) like premature convergence, multipopulation genetic algorithm (MPGA) is proposed to improve the algorithm efficiency. The logic diagram of the algorithm is shown in Figure 2.

#### 4.2.3. Comparison of SGA and MPGA Performance

As shown in Table 6, MPGA has better performance in algorithm stability and more powerful ability of global search than SGA.

### 4.3. Sensitivity Analysis

#### 4.3.1. Arrival Rate

**Figure 3:**
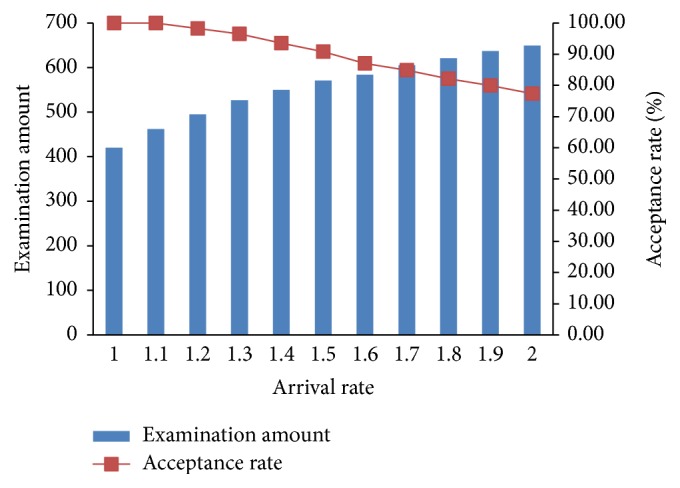
Arrival rate and acceptance rate.

**Figure 4:**
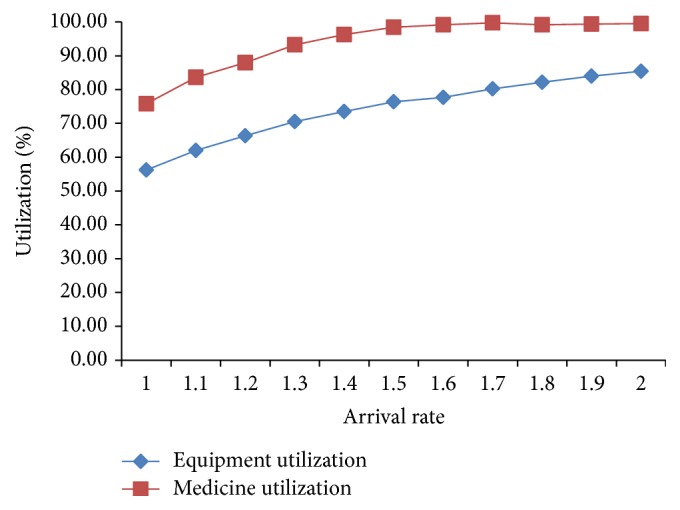
Arrival rate and utilization.

We study the influence of increased demand on outputs which are shown in Figures 3 and 4.

As seen in Figure 3, with the arrival rate gradually doubling its original one, total examination amount is rising slowly and flattens in the end which indicates the maximum utilization of resources. Acceptance rate is the percentage of accepted patients to patients' arrivals in a week. It decreases with the increasing of the arrival rate at an increasing rate because of resource constraints. When the arrivals attain a certain degree, a higher proportion of patients are refused.

As seen in Figure 4, equipment and medicine utilization rates tend to reach an equilibrium situation after experiencing a slow increase in the arrival rate. The medicine utilization curve is above the equipment one, which indicates that the tighter resource is medicine.

**Figure 5:**
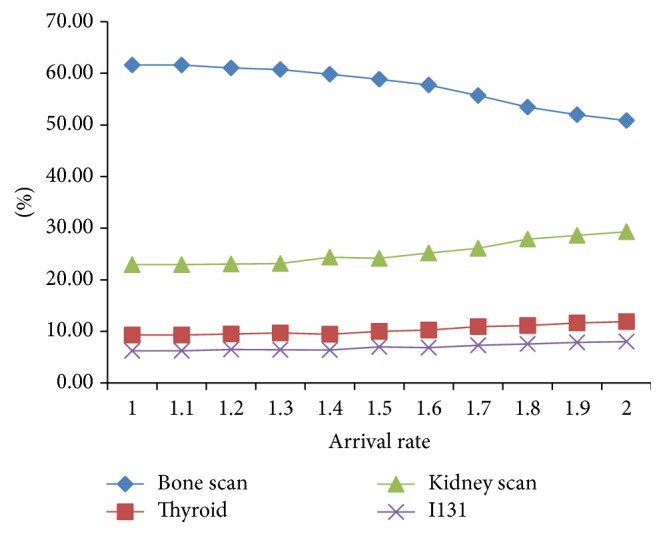
Arrival rate and acceptance proportion of each item.

**Figure 6:**
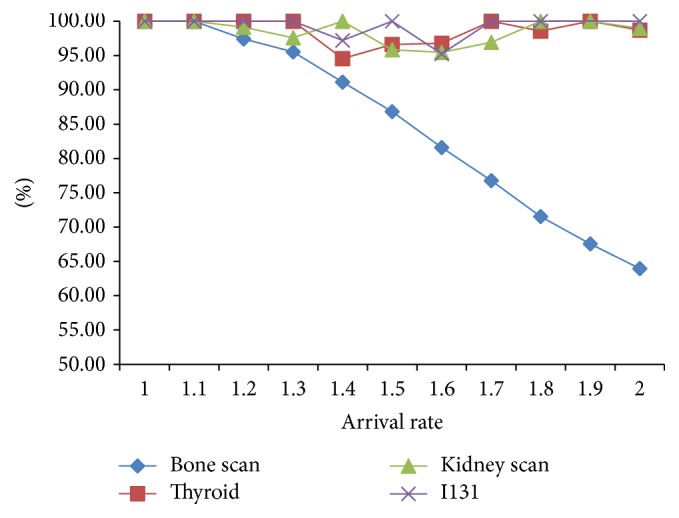
Arrival rate and acceptance rate of each item.

As seen in Figure 6, the acceptance rate of bone scan decreases from 100% to 63.95% with the increasing of arrival rate, while the rest stays above 95% mainly because the bone scan is the biggest resource consumer (see Figure 5). It also shows that the equipment and medicine resources can only meet 1.1 times the demand. That is to say, when the arrival rate is within 1.1 times, all patients are accepted; otherwise, the acceptance rates of every item start to fall.

#### 4.3.2. Equipment and Medicine Constraints

Being two key constraints in scheduling, the equipment and medicine constraints are studied for how their changes might affect the target value. The equipment working time is set at 8 to 12 hours every half hour and the medicine production is set at 80% to 120% of the original production every 5%. Since there are nine equipment constraint values and nine medicine constraint values, the combination of both has 81 values.

**Figure 7:**
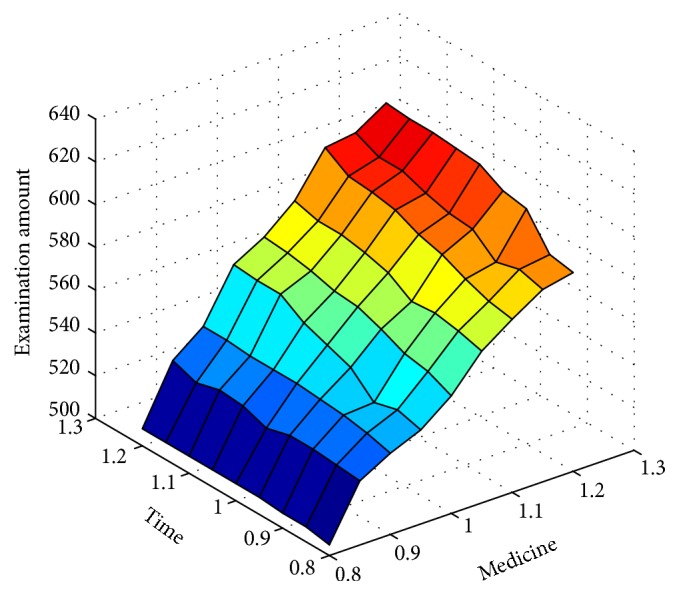
Sensitivity analysis of resource constraints.

Figure 7 shows that the target value gradually increases with broadening constraints of equipment (working time) and medicine (production), but the sensitive effect of the medicine production change on the target value is greater than that of the equipment working time, which also indicates that the tighter resource is medicine.

## 5. Online Scheduling Model

### 5.1. Online Schedule Strategy

The online schedule strategy mainly aims at adjusting the daily amount of each item to maximize the resource utilization. Three online scheduling strategies together with the current one are compared and analyzed to obtain the optimum solution. In addition, for the offline optimal scheduling programming rule, hereinafter referred to as “Offline” for short, being an ideal one (because, in the evaluation stage, the arrival sequence is known), it is added as the highest standard.

**Strategy 1 (current).**

This is the current scheduling strategy (see Table 7).

**Strategy 2 (FCFS).**

Making no reservation for any items, scheduling is based on constraints of equipment and medicine with FCFS discipline.

**Strategy 3 (Offopt).**

This is the offline optimal scheduling strategy solved by MPGA based on history data (see Table 8).

**Strategy 4 (dynamic).**

This is the optimal dynamic scheduling strategy.

**Figure 8:**
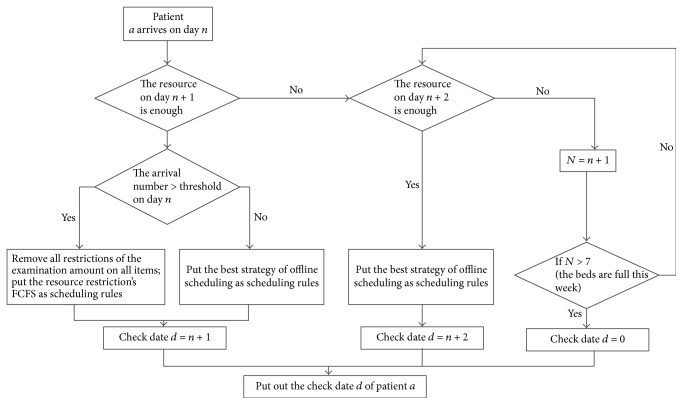
The logic diagrams of Strategy 4.

Strategy 3 based on history data cannot ensure the optimal online results due to the randomness of the daily arrival rate and arrival sequence. Therefore, we design Strategy 4 which specifies that when the arrivals reach the threshold level while the resource becomes surplus the next day, the scheduling rule shifts from Strategy 3 to Strategy 2. The logic diagram of Strategy 4 is shown in Figure 8.

### 5.2. Simulation Results Analysis

#### 5.2.1. Target Value

**Figure 9:**
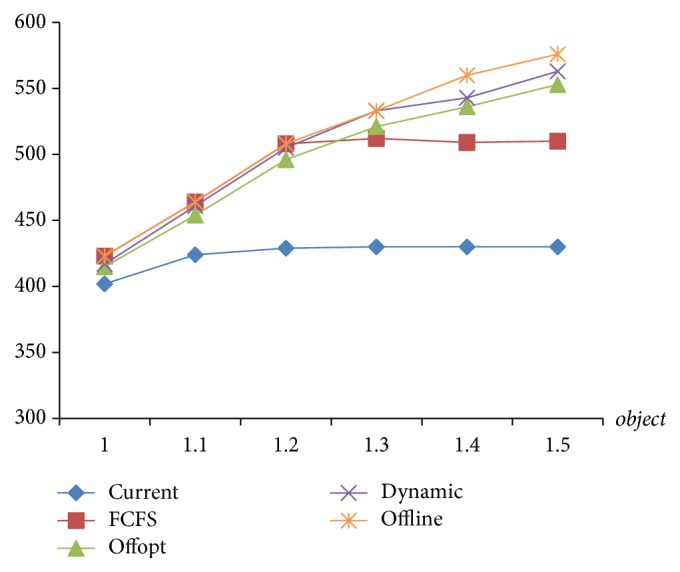
The comparison of target value of 4 strategies.

The target value is the weekly examination amount which is the key indicator of the strategy assessments. As seen in Figure 9, Strategy 1 is always the worst. The performance of Strategy 2 is consistent with that of “Offline” before the arrival rate reaches 1.2 times, but after 1.3 times, it declines markedly and is only slightly better than Strategy 1. Strategy 3 is always lower than Strategy 4 which is very close to “Offline.” Although Strategy 4 has some difference with “Offline” after the arrival rate reaches 1.3 times, it is far better than the other three. Therefore, Strategy 4 is the optimal one.

#### 5.2.2. Utilization of Equipment and Medicine

**Figure 10:**
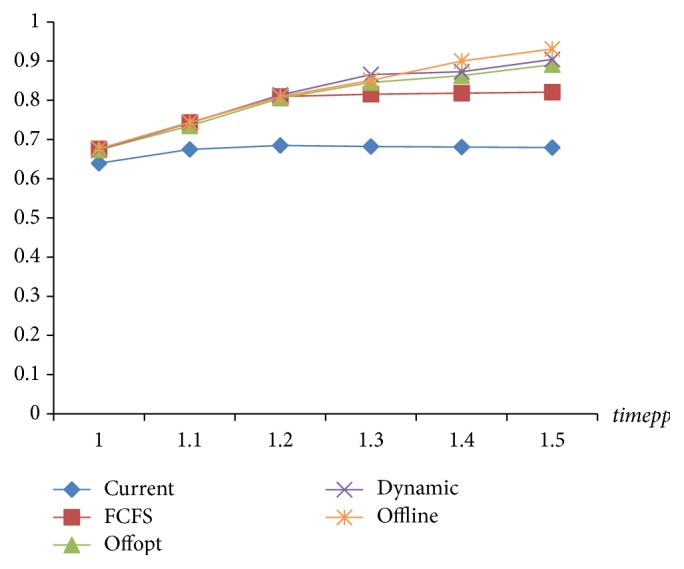
The comparison of equipment utilization of 4 strategies.

**Figure 11:**
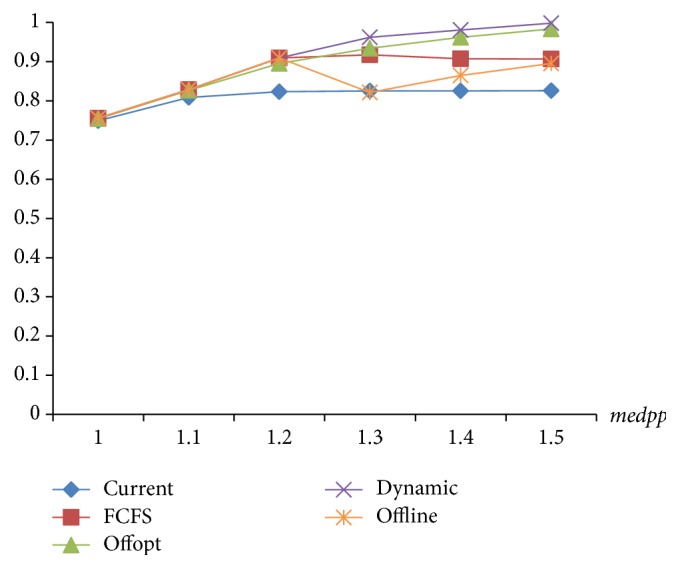
The comparison of medicine utilization of 4 strategies.

Due to the distribution of examination items, strategies with the same target value will have different utilization of equipment and medicine. The essential characteristic of optimization is to improve resource utilization. Therefore, the utilization of equipment and medicine is another indicator in strategy assessment. As seen in Figures 10 and 11, Strategy 4 is the best of the four in utilization of equipment and medicine.

#### 5.2.3. Acceptance Rate and Timely Rate

Patients who cannot get an appointment within a week (the scheduling period) are regarded as being refused. Therefore, the acceptance rate, the percentage of received patients to patients' arrivals in a week, measures the ability to satisfy time window constraints. However, even when people get an appointment during the scheduling period, their examination date may not be within the time window. Therefore, timely rate, the percentage of patients getting an appointment within the time window to received patients, is another index for estimating the ability to satisfy time window constraints.

**Figure 12:**
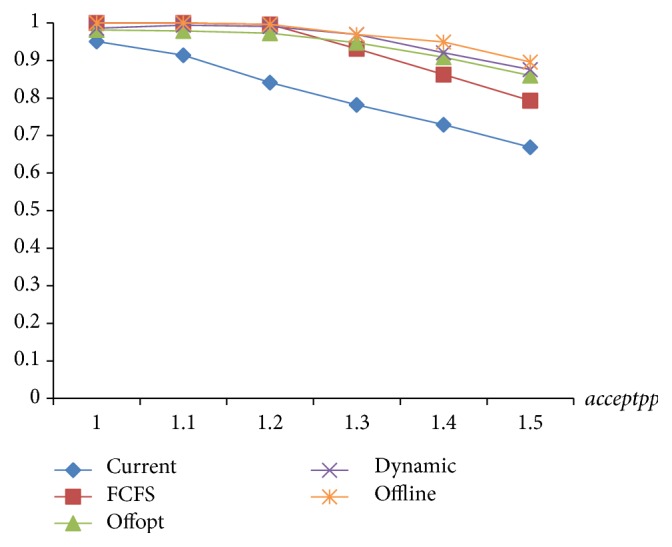
Comparison of the acceptance rate of 4 strategies.

**Figure 13:**
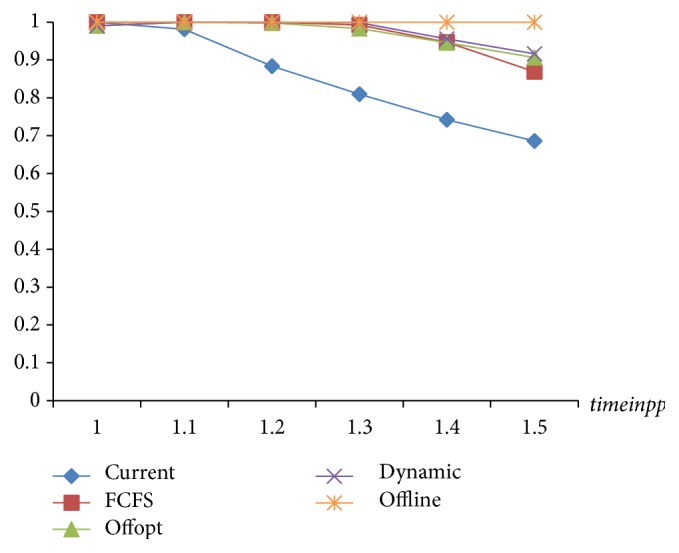
Comparison of the timely rate of 4 strategies.

As seen in Figures 12 and 13, constrained by the time window, “Offline” has the highest acceptance rate and 100% timely rate. Strategy 4 is marginally under the “Offline” but better than the others, followed by Strategies 3 and 2. The current strategy is the worst.

### 5.3. Strategy Stability Analysis

#### 5.3.1. The Random Arrival Sequence

The data in this paper contains information of arrival date without exact arrival time, so the daily arrivals of each item are known and the arrival sequence is unknown. Considering weak statistical regularity of real arrival sequence, we generate arrival sequence by random method based on history data. Then, the effect of the random arrival sequence on output result is analyzed. To be more detailed, 10 arrival sequences are generated with the daily arrivals of each item group being fixed and the effects on the target value of the four strategies are compared under two supply and demand situations.

**Figure 14:**
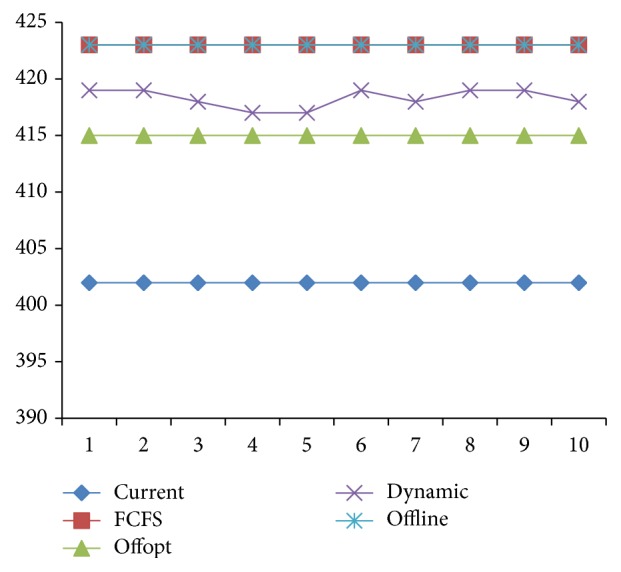
Arrival sequence random testing in the oversupply situation.

**Figure 15:**
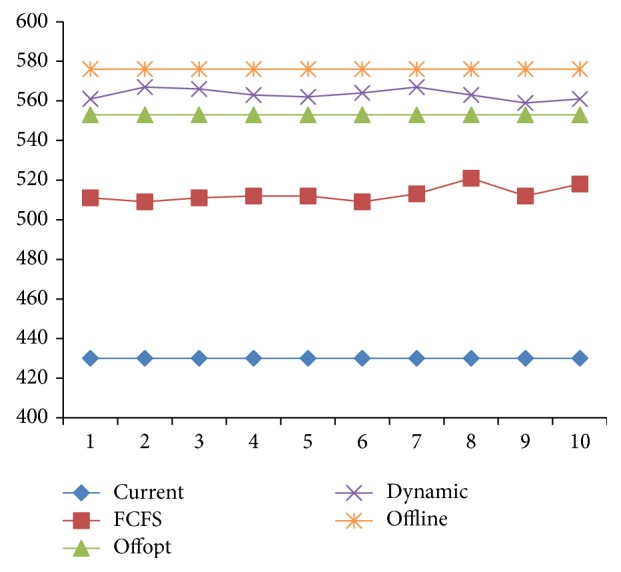
Arrival sequence random testing in the undersupply situation.

As shown in Figure 14, in the oversupply situation, the curve of Strategy 2 overlaps with that of “Offline” and only Strategy 4 is affected by the randomness of the arrival sequence; in the undersupply situation (see Figure 15), both Strategies 2 and 4 are affected. However, the rank of strategy is not affected.

#### 5.3.2. The Randomness of the Arrival Rate

As mentioned in Section 4, most data of arrival obey Poisson's distribution and the randomness of arrival rate may have a certain impact on output in the online scheduling. Furthermore, the performances of strategies vary with different demands. Therefore, the randomness of the arrival rate is tested under two supply and demand situations, respectively. In the oversupply situation the arrival rate is set to 1 time, and in the undersupply situation it is set to 1.5 times. In both situations, the simulation tests for the 4 strategies are conducted 10 times.

**Figure 16:**
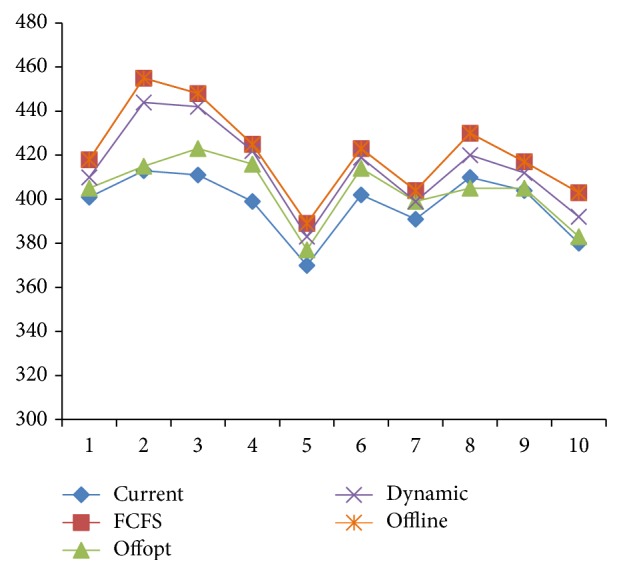
Arrival rate random testing in the oversupply situation.

Figure 16 shows that each strategy is affected by the randomness of arrival rate to a certain extent when there is less demand. But Strategy 2 always coincides with the offline optimal scheduling rules, which shows that, in the oversupply situation, Strategy 2 can achieve the optimal value. Strategy 4 which in most cases coincides with “Offline” comes second and is much better than Strategies 1 and 3. Strategy 1 is always the worst.

**Figure 17:**
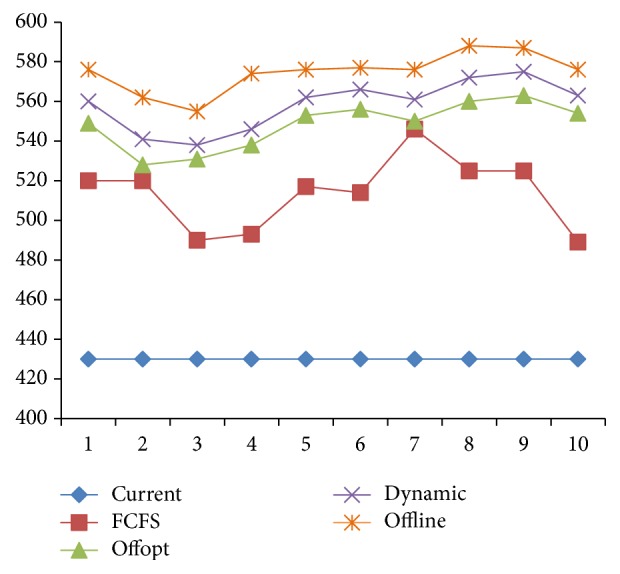
Arrival rate random testing in the undersupply situation.

Figure 17 shows that, in the undersupply situation, Strategy 1 can only receive a fixed number of patients and does not have fluctuations with the randomness of arrival rate for being artificially designed and not making full use of resources; the randomness of arrival rate has higher influence on Strategy 2 which is only preferable over Strategy 1. Though Strategy 4 fails to reach “Offline,” it has optimal result and is less affected by the randomness.

### 5.4. Discussion

From the above analysis, we can see that, in the current situation, the gap between Strategy 1 and the others is not obvious owing to the hospital having already got a good scheduling strategy tailored to its circumstances. However, as the simulation has shown, the weekly examination amount and the utilization of equipment and medicine will not increase with demand, which is growing at a rate of nearly 20% a year in the WCH, under this strategy, which will lead to sharp declines in the acceptance rate and timely rate, resulting in supply and demand contradiction. To solve this problem, there are two major methods. One is to invest more resources and the other is to maximize resource utilization. In view of heavy spending on expensive equipment and other resources, it might be more effective and economical to optimize scheduling strategy.

Strategy 2 uses FCFS rule, which specifies that a job with the longest waiting time in the ready queue containing jobs that are ready to run should be executed first. The advantages of this algorithm are simple in theory and easy to accomplish. On the face of it, it might seem like a fair rule, but essentially it is not for quite a few short run-time jobs needing to wait for a long time for an earlier started long run-time job. Obviously, it is more favorable than the latter. In less resource-critical situations, Strategy 2 can still have good performance. Simulation results show that the weekly examination amount, the utilization of equipment and medicine, acceptance rate, and timely rate are all approximate to those of “Offline” before the arrival rate reaches 1.2 times. But with severe shortage of key resources, the influence from the long run-time job using a large quantity of resources becomes preeminent, resulting in bad strategy performance. As the simulation has shown, after the arrival rate reaches 1.2 times, it becomes inferior to “Offline” and above 1.3 times it declines markedly with the weekly examination amount, acceptance rate, and timely rate being the second lowest. Besides, FCFS rule is easily affected by many random factors. Therefore, FCFS rule is not appropriate to be used separately and should be combined with other rules in scheduling strategy.

Strategy 3 is more scientific and complicated than the current one. It is based on the real data collected from the Nuclear Medicine Department of the WCH and is an offline optimum value solved by MPGA after systematically analyzing the status quo, such as the examination flow, the resource constraints, and current strategy. Unlike all the examination items that are only roughly split into two categories in the current strategy, the top 8 examination items, making up 96.59% of the total amount, are focused on and further divided into four groups based on similar resources consumption in Strategy 3. However, Strategy 3 cannot ensure the online optimization result due to the randomness of daily arrival rate and arrival sequence. For instance, restricted by Strategy 3, even when a certain type of patient arrival is insufficient the next day, spare resources cannot be allocated to other types of patients, which leads to serious resource waste.

To solve this problem, Strategy 4 is designed to make our scheduling strategy dynamically adjust to actual patient arrivals, which specifies that when the arrivals reach the threshold level while the resource becomes surplus the next day, the rule shifts from Strategy 3 to FCFS rule. Such dynamic scheduling strategy is widely used in various areas. In airline revenue management [24–26], for example, airlines are generally divided into first class, business class, and economy class with a constant proportion. If economy-class seats have been sold out with only first- or business-class seats available shortly before takeoff, the first- or business-class ticket will be reduced, costing the same as an economy-class ticket to better utilize resources and maximize the overall operational revenue. Among four scheduling strategies, Strategy 4 based on offline optimal dynamic scheduling strategy has the best performance and has a high stability. As shown in Table 9, the examination amount increases by 29.76% compared with the current one with a significant increase in the resource utilization and timely rate. In addition, this strategy bears the advantage of convenient and economic operation. Pérez et al. [1] derived a complicated stochastic online scheduling algorithm considering the human resources (technologists, nurses), equipment (gamma cameras or a treadmill), and radiopharmaceuticals in more than 60 procedures in nuclear medicine. Accurate algorithm as it is, it should be implemented in JAVA and ILOG CPLEX. In this paper, however, only two key resources are considered based on four examination item groups and FCFS rule is applied when the arrivals reach the preset point, which enable the practicer, usually nurse in the nuclear medicine department, with limited computer operation skills to do the job. Therefore, it is perhaps a good tradeoff between the feasibility and scientificity.

## 6. Conclusions

Due to the special characteristics of multiresources and time window constraints, scientific and reasonable appointment scheduling in the nuclear medicine department is not an easy task. In this paper, a stochastic online appointment schedule strategy is derived considering multiresources allocation, the time window constraints imposed by short-lived radiopharmaceuticals, and the stochastic nature of the patient requests. A series of experiments are conducted to show the effectiveness of the proposed strategy based on data provided by the WCH. The results show that the examination amount increases by 29.76% compared with the current one with a significant increase in the resource utilization and timely rate. Besides, it also has a high stability for stochastic factor and bears the advantage of convenient and economic operation. Although the study and the related results are based on the nuclear medicine department, the online appointment scheduling system can play a reference and instructive role for the other radiology departments. There are some limitations in the present study which has a wide range of the time window (1–3 days), hoping that the research can get a more accurate scheduling strategy by refining the time window constraints in the next step.

## Figures and Tables

**Table 1:** Source of medicine.

Medicine	Half-life	Source	Comments
Iodine-131 (I131)	8.3 days	Outsourcing	Purchased directly from the market
Technetium-99 (Tc99)	6.02 hours	Self-manufacturing	Generated by centrifugal accelerator

**Table 2:** The resource consumption of item groups.

Item groups	Procedure time ①	Medicine type ②	Dosage ③	Injection method ④	③/①
Group 1 Whole body bone scan Bone dynamic scan	18 min	Tc	30 mCi	Nonbedside	1.67
Group 2 Thyroid scan	4 min	Tc	2-3 mCi	Bedside	0.5
Group 3 Salivary glands scan Kidney scan GFR	35 min	Tc	5 mCi	Bedside	0.15
Group 4 I131 therapeutic agent scan I131 whole body scan	12 min	I	*∗*	Per os	0

*Preparation Time*. 30 s for getting in and out of bed, 1–3 min for bedside injection.

**Table 3:** Weekly medicine production.

	Mon.	Tues.	Wed.	Thur.	Fri.	Sat.	Sun.
Production (unit: mCi)	2900	2140	1660	1450	1300	990	620

**Table 4:** Arrival distribution of four item groups in one week.

	Group 1	Group 2	Group 3	Group 4
Mon.	Poisson (43)	Poisson (12)	Poisson (15)	Uniform (0, 2)
Tues.	Poisson (49)	Poisson (11)	Poisson (20)	Poisson (4)
Wed.	Poisson (53)	Poisson (8)	Poisson (21)	Poisson (5)
Thur.	Poisson (50)	Poisson (4)	Poisson (18)	Poisson (5)
Fri.	Poisson (41)	Uniform (0, 3)	Poisson (13)	Poisson (8)
Sat.	Poisson (13)	Uniform (0, 1)	Poisson (5)	Poisson (3)
Sun.	Poisson (11)	Poisson (3)	Poisson (3)	Uniform (0, 1)

**Table 5:** The results of the genetic algorithm.

Population size	Generation	Crossover probability	Mutation probability	Operation time	Optimum
700	3000	0.9	0.2	721 s	576

**Table 6:** Performance comparison of SGA and MPGA.

	Mean	Median	Standard deviation	Standard error	Range	Min.	Max.
SGA	567.8	566	8.01249	3.58329	18	558	576
MPGA	576.2	577	1.09545	0.4899	2	575	577

**Table 7:** Scheduling rule of Strategy 1.

Item	Mon.	Tues.	Wed.	Thur.	Fri.	Sat.	Sun.
Bone scan (whole body bone/bone dynamic scan)	70	60	50	43	35	30	0
Others	15	20	22	20	15	25	25

**Table 8:** Scheduling rule of Strategy 3.

Item groups	Mon.	Tue.	Wed.	Thur.	Fri.	Sat.	Sun.
Group 1	72	57	59	50	47	15	10
Group 2	5	9	15	5	3	2	0
Group 3	6	22	18	20	0	26	17
Group 4	0	3	5	9	18	5	1

**Table 9:** Comparison of Strategies 1 and 4.

Situation	Strategy	Examination amount	Equipment utilization	Medicine utilization	Acceptance rate	Timely rate
Oversupply	1	402	63.90%	74.92%	95.04%	100.00%
	4	417	67.40%	75.50%	98.58%	99.04%
Undersupply	1	430	67.96%	82.59%	66.87%	68.60%
	4	563	90.44%	99.84%	87.56%	91.65%

## Scheduling Problem Sets and Parameters

*G*: Set of examination items, indexed *g*
*P*: Set of patients, indexed *p*
*D*: Set of working days, indexed *d*
*t*_*g*_: Examination period of item group *g*
*k*_*g*_: Dose of item group *g*
*t*_*d*_: Working time of day *d*
*k*_*d*_: Medicine production of day *d*
*w*_*l*_: Lower limit of time window
*w*_*u*_: Upper limit of time window
*x*_*gpd*_: Whether patient *p* of item group *g* is examined on day *d*
*a*_*gp*_: Patient *p* of arrival day *g*.

## References

[1] Pérez E., Ntaimo L., Malavé C. O., Bailey C., McCormack P. (2013). Stochastic online appointment scheduling of multi-step sequential procedures in nuclear medicine. *Health Care Management Science*.

[2] Gupta D., Denton B. (2008). Appointment scheduling in health care: challenges and opportunities. *IIE Transactions*.

[3] Deceuninck M., Fiems D., De Vuyst S. (2018). Outpatient scheduling with unpunctual patients and no-shows. *European Journal of Operational Research*.

[4] Lim C., Chodhari R. (2017). Re: a dynamic approach for outpatient scheduling. *Journal of Medical Economics*.

[5] Zhu H., Hou M., Wang C., Zhou M. (2012). An efficient outpatient scheduling approach. *IEEE Transactions on Automation Science and Engineering*.

[6] Leeftink G., Hans E. W. (2018). Case mix classification and a benchmark set for surgery scheduling. *Journal of Scheduling*.

[7] Luo L., Luo Y., You Y., Cheng Y., Shi Y., Gong R. (2016). A MIP model for rolling horizon surgery scheduling. *Journal of Medical Systems*.

[8] Marques I., Captivo M. E., Vaz Pato M. (2015). A bicriteria heuristic for an elective surgery scheduling problem. *Health Care Management Science*.

[9] Jia-Qi Y. I., Zhou L. P., Geng N. (2017). A stochastic programming based approach for perinatal examination appointment scheduling optimization. *Industrial Engineering and Management*.

[10] Qiu H., Wang D., Wang Y., Yin Y. (2017). MRI appointment scheduling with uncertain examination time. *Journal of Combinatorial Optimization*.

[11] Saharan S., Kumar R. (2016). Graph coloring based optimized algorithm for resource utilization in examination scheduling. *Applied Mathematics & Information Sciences*.

[12] Cayirli T., Veral E. (2003). Outpatient scheduling in health care: a review of literature. *Production Engineering Research and Development*.

[13] Green L. V., Savin S. V., Wang B. (2006). Managing patient service in a diagnostic medical facility. *Operations Research*.

[14] Patrick J., Puterman M. L., Queyranne M. (2008). Dynamic multipriority patient scheduling for a diagnostic resource. *Operations Research*.

[15] Kolisch R., Sickinger S. (2008). Providing radiology health care services to stochastic demand of different customer classes. *OR Spectrum*.

[16] Wu X., Li J., Xu R., Yu T. A simulation study of appointment scheduling for multi-class MRI examination.

[17] Akhavizadegan F., Ansarifar J., Jolai F. (2017). A novel approach to determine a tactical and operational decision for dynamic appointment scheduling at nuclear medical center. *Computers & Operations Research*.

[18] Sauré A., Patrick J., Tyldesley S., Puterman M. L. (2012). Dynamic multi-appointment patient scheduling for radiation therapy. *European Journal of Operational Research*.

[19] Liu Y., Geng N. (2017). Outpatient scheduling for multiple examinations. *Operations Research & Management Science*.

[20] Karhi S., Shabtay D. (2014). Online scheduling of two job types on a set of multipurpose machines. *International Journal of Production Economics*.

[21] Ma R., Tao J., Yuan J. (2016). Online scheduling with linear deteriorating jobs to minimize the total weighted completion time. *Applied Mathematics and Computation*.

[22] Ni G. Q., Xu Y. F. (2011). Competitive analysis of dynamic online booking policies in revenue management. *Systems Engineering-Theory and Practice*.

[23] Ball M. O., Queyranne M. (2009). Toward robust revenue management: competitive analysis of online booking. *Operations Research*.

[24] Gosavii A., Bandla N., Das T. K. (2002). A reinforcement learning approach to a single leg airline revenue management problem with multiple fare classes and overbooking. *Institute of Industrial Engineers (IIE). IIE Transactions*.

[25] Ni G., Xu Y. (2012). Online joint pricing and booking policies in airline revenue management. *Combinatorial optimization and applications*.

[26] Jiang H., Miglionico G. (2014). Airline network revenue management with buy-up. *Optimization*.

